# Predictors for presence and abundance of small mammals in households of villages endemic for commensal rodent plague in Yunnan Province, China

**DOI:** 10.1186/1472-6785-8-18

**Published:** 2008-12-10

**Authors:** Jia-Xiang Yin, Alan Geater, Virasakdi Chongsuvivatwong, Xing-Qi Dong, Chun-Hong Du, You-Hong Zhong, Edward McNeil

**Affiliations:** 1 Yunnan Institute of Endemic Disease Control and Prevention, 5 Wenhua Road, Dali City, Yunnan Province, 671000, PR China; 2 Epidemiology Unit, Faculty of Medicine, Prince of Songkla University, Hat Yai, Songkhla 90112, Thailand

## Abstract

**Background:**

Ninety-one rodent plague epidemics have occurred in Lianghe county, Yunnan Province, China, between 1990 and 2006. This study aimed to identify predictors for the presence and abundance of small mammals in households of villages endemic for rodent plague in Lianghe county.

**Results:**

*Rattus flavipectus *and *Suncus murinus *were the two species captured in 110 households. Keeping cats decreased the number of captures of *R. flavipectus *by one to two thirds and the chance of reported small mammal sightings in houses by 60 to 80%. Food availability was associated with fewer captures. Keeping food in sacks decreased the small mammal captures, especially of *S. murinus *4- to 8-fold. Vegetables grown around house and maize grown in the village reduced the captures of *S. murinus *and *R. flavipectus *by 73 and 45%, respectively. An outside toilet and garbage piles near the house each reduced *R. flavipectus *captures by 39 and 37%, respectively, while raising dogs and the presence of communal latrines in the village increased *R. flavipectus *captures by 76 and 110% but were without detectable effect on small mammal sightings. Location adjacent to other houses increased captures 2-fold but reduced the chance of sightings to about half. In addition, raising ducks increased the chance of sighting small mammals 2.7-fold. Even after adjusting for these variables, households of the Dai had higher captures than those of the Han and other ethnic groups.

**Conclusion:**

Both species captures were reduced by availability of species-specific foods in the environment, whereas other predictors for capture of the two species differed. Other than the beneficial effect of cats, there were also discrepancies between the effects on small mammal captures and those on sightings. These differences should be considered during the implementation and interpretation of small mammal surveys.

## Background

Pneumonic plague epidemics in early 20th century China killed tens of thousands of persons [1]. Plague is consequently ranked first among all communicable diseases regulated by *The Law for Communicable Disease Control and Prevention *in China. The "Third Pandemic" began in Yunnan Province, China in 1855, and it is thought to be still ongoing [2,3]. Human plague outbreak has occasionally occurred in this province [4]. Yunnan is the most serious province in China regarding plague epidemics. Currently, about 10 million people live in plague endemic areas in Yunnan. Plague is regarded as an important public health problem in the province [5].

Plague is a zoonosis that primarily affects rodents. This disease is driven by the rodent population and rising rodent numbers increase the chance of an outbreak [6,7]. This is because high abundance may lead not only to more contact between humans and rodents, but also to outbreaks within the reservoir population [8]. If the rodents are kept at a permanently low level, then the risk of a large outbreak in rodents, and therefore the risk of human plague, will be reduced [6].

Many factors, such as rodent density and species, flea index and species, plague bacteria, climate, and environment, may influence the dynamics of enzootic plague cycles [9-12]. Among them, the abundance of rodents and fleas are two main influencing factors – proximate determinants for plague emergence. Rat and flea control are also the most common techniques for controlling this disease and are very useful and important for preventing the spread of plague [8].

While the population of small mammals is directly determined by births, deaths, immigration and emigration [13], extraneous factors related to human eco-behavior in endemic areas of commensal rodent plague are not well documented. To improve plague prevention and control programs in these areas, there is a clear need for a better understanding of these determinants of the population size of small mammals in plague foci. The objective of the present study was to identify predictors for the presence and abundance of small mammals in houses of villages endemic for commensal rodent plague in Lianghe county.

## Results

The characteristics of the 600 study households are shown in Table 1. Seventy five percent of the households belonged to the Dai ethnic group, 97% were farming families, and 69% had an annual family income of less than 8,000 RMB. The median number of family members was 5 persons, and the highest education level among family members was primary school or lower secondary school in 78% of households.

**Table 1:** Characteristics of the study households.

**Interviewing variable**	n*	**%**	**Interviewing variable**	n*	**%**	**Observing variable**	n*	**%**
**Economy source-planting**			**Animal keeping in house**			**House construction**		
No	12	2.0	No	28	4.7	Earth and wood	523	87.4
Yes	585	97.5	Yes	572	95.3	Brick and wood	74	12.4
**Annual income **(RMB)			**Keeping cat**			**Surroundings – field**		
<4000	167	27.8	No cat	218	36.3	No	281	46.8
4000–8000	249	41.5	Sleep in house	301	50.2	Yes	319	53.2
8000–12000	90	15.0	Not sleep in house	54	9.0	**Surroundings – house**		
>12000	93	15.5	**Keeping dog**			No	81	13.5
**Education level**			No	347	57.8	Yes	519	86.5
Primary school	155	25.8	Yes	225	37.5	**Surroundings – road**		
Middle school	314	52.3	**Keeping chicken**			No	502	83.7
High school	76	12.7	No	138	23.0	Yes	98	16.3
Other	50	8.9	Yes	434	72.3	**Surroundings – canal**		
**Number of family members**			**Keeping pig**			No	258	43.0
<4	85	14.2	No	159	26.5	Yes	342	57.0
4~5	342	57.0	Yes	413	68.8	**Crops grown near house**		
>5	172	28.7	**Keeping cattle**			No	146	24.3
**Ethnic group**			No	40	6.7	Yes	453	75.5
Han and other	131	21.8	Yes	246	41.0	**Vegetable grown near house**		
Dai	468	78.0	**Keeping duck**			No	154	25.7
**Drinking water**			No	255	42.5	Yes	299	49.8
Well	36	6.0	Yes	31	5.2	**Fruit grown near house**		
Piped water	559	93.2	**Seeing rat in houses**			No	134	22.3
**Store food**			No	28	4.7	Yes	319	53.2
No	25	4.2	Yes	379	63.2	**Maize grown near house**		
Yes	564	94.0	**Seeing rat in crops**			No	390	65.0
**Type of food storage**			No	227	37.8	Yes	62	10.3
Sack	168	29.2	Yes	182	30.3	**Paddy grown near house**		
Metal drum	60	10.4	**Rat problem in house**			No	331	55.2
Wood drum	145	25.2	No	378	63.0	Yes	121	20.2
Other	196	25.2	Yes	218	36.3	**Sugarcane grown near house**		
**Waste disposal**			**Seeing rat faeces in house**			No	352	58.7
Put in garden	36	6.0	No	369	61.5	Yes	99	16.5
Put in canal	404	67.3	Yes	231	38.5	**Rubbish dump near house**		
Other	155	25.8	**Rat control**			No	308	51.3
**Toilet**			No	132	22.0	Yes	291	48.5
No toilet	345	57.7	Yes	468	78.0	**Observing rat faeces in house**		
Inside of house	43	7.1	**Frequency of rat control**			No	509	84.8
Outside of house	211	35.2	Once a year	92	15.3	Yes	89	14.8
**Type of toilet**			Once half a year	308	51.3			
Water sealed	15	5.9	Once a month	31	5.2			
Semi-open and open	239	93.7	Other	35	5.8			

* Total number of households may be less than 600 in some variables because of missing values.

A total of 166 small mammals were caught in 110 households (18%). They comprised 2 species, namely the rodent, *Rattus flavipectus *(133 individuals, or 80% of all trapped small mammals, from 87 households) and the insectivore, *Suncus murinus *(33 individuals, or 20% of all trapped mammals, from 26 households). Both species were caught in 3 households. There was no association between the two species in the same household (p = 1, Fisher test). One animal was captured in each of 74 households, and more than one (2~5) in 36 households.

Of the 598 households that responded to the question about seeing "rats" in their house, 380 (63.5%) reported the sighting of small mammals in houses within the previous 2 weeks. Eighty-one (21.3%) of these households had animal captures compared to 29 among 218 households (13.3%) not seeing small mammals (p = 0.02, chi-square test).

Table 2 shows the distribution of numbers of household captures of small mammals, *R. flavipectus *and *S. murinus*, and reported household sightings of small mammals within the last 2 weeks, according to household-level variables that indicated some evidence of predictive ability (p < 0.2) for at least one of the outcomes as revealed by the univariate analysis techniques mentioned above. None of the univariate Poisson models showed any evidence of violation of the Poisson assumption. Finally, 12 household-level variables were entered into the prototype model of total small mammal captures, 10 variables into the prototype model for *R. flavipectus *captures, 7 variables into the prototype model of *S. murinus *captures, and 10 variables into the prototype model of sightings or capture of small mammals.

**Table 2:** Distribution of total small mammal, *R. flavipectus *and *S. murinus *household captures, and reported household sightings or capture of small mammals, according to variables considered as initial candidate variables for initial multivariate modelling.

	**Total small mammal captures^a^**				***R. flavipectus *captures^a^**				***S. murinus *captures^a^**			**Small mammal presence^b^**		
				
**Variable**	**0**	**1**	**2~5**	**P^c^**	**0**	**1**	**2~5**	**P^c^**	**0**	**1~3**	**P^c^**	**No**	**Yes**	**P^d^**
**Ethnic group**				0.025				0.023			0.640			0.643
Han and other	130	13	5		134	10	4		143	5		44	104	
Dai	360	61	31		379	46	27		431	21		145	305	
**Type of food storage**				0.006				0.004			0.009			0.308
Sack	140	19	9		142	17	9		166	2		51	117	
Metal drum	44	9	7		47	7	6		56	4		14	46	
Wood drum	115	20	10		120	16	9		139	6		44	100	
Other	191	26	10		204	16	7		213	14		80	146	
**Keeping cat**				0.008				0.006			0.351			<0.001
No cat	166	34	18		177	25	16		205	13		40	178	
Sleep in house	252	33	15		263	24	14		291	10		114	185	
Not sleep in house	47	5	2		49	4	1		52	2		26	28	
Missing	24	2	1		24	3	0		26	1		9	18	
**Keeping dog**				0.043				0.001			0.004			0.091
No	311	46	18		329	33	13		355	20		128	246	
Yes	179	28	18		184	23	18		219	6		61	163	
**Keeping chicken**				0.168				0.237			0.475			0.436
No	131	22	13		137	19	10		158	8		48	118	
Yes	359	52	23		376	37	21		416	18		141	291	
**Keeping cattle**				0.010				0.003			0.868			0.186
No	279	51	24		291	42	21		339	15		104	250	
Yes	211	23	12		222	14	10		235	11		85	159	
**Keeping duck**				0.626				0.250			0.061			0.191
No	465	72	32		488	54	27		543	26		183	384	
Yes	25	2	4		25	2	4		31	0		6	25	
**House construction**				0.275				0.517			0.230			0.143
Earth and wood	426	67	33		447	51	28		502	24		160	364	
Brick and wood	64	7	3		66	5	3		72	2		29	45	
**Surroundings – house**				0.010				0.060			0.038			0.068
No	74	5	2		75	4	2		80	1		18	63	
Yes	416	69	34		438	52	29		494	25		171	346	
**Surroundings – canal**				0.682				0.401			0.438			0.153
No	207	34	17		217	26	15		247	11		73	185	
Yes	283	40	19		296	30	16		327	15		116	224	
**Vegetable grown near house**				0.009				0.206			<0.001			0.774
No	234	45	22		250	34	17		282	19		93	208	
Yes	256	29	14		263	22	14		292	7		96	201	
**Fruit grown near house**				0.414				0.960			0.052			0.126
No	224	39	18		237	31	13		266	15		98	183	
Yes	266	35	18		276	25	18		308	11		91	226	
**Maize grown near house**				0.010				0.014			0.386			0.237
No	435	68	35		456	52	30		514	24		174	362	
Yes	55	6	1		57	4	1		60	2		15	47	
**Sugarcane grown near house**				0.038				0.148			0.069			0.447
No	402	66	33		423	50	28		477	24		154	345	
Yes	88	8	3		90	6	3		97	2		35	64	
**Waste disposal**				0.888				0.764			0.668			0.048
Put in garden	29	5	2		30	4	2		35	1		13	23	
Put in canal	330	48	26		346	37	8		386	18		114	288	
Other	131	21	8		137	15	8		153	7		62	98	
**Rubbish dump near house**				0.010				0.011			0.484			0.617
No	243	43	23		256	32	21		294	15		94	214	
Yes	247	31	13		257	24	10		280	11		95	195	
**Location of toilet**				0.003				0.007			0.395			0.053
No toilet	275	44	27		289	34	23		329	17		97	247	
Inside house	34	5	4		37	2	4		40	3		19	24	
Outside house	181	25	5		187	20	4		205	6		73	138	

^**a **^Total number of households is 600.

^**b **^Total number of households is 598.

^**c **^P value from likelihood ratio test in univariate Poisson regression model.

^**d **^P value from chi square test.

Table 3 shows the distribution of total small mammal captures per village and total households with small mammal captures per village. Based on univariate Poisson regression models and likelihood ratio tests (p < 0.2), seven candidate village-level variables were selected for inclusion as candidate variables in initial multilevel analyses together with household-level variables.

**Table 3:** Distribution of total captures and number of households with captures per village, according to variables considered as candidate variables for initial multilevel modelling.

		**Total small mammal captures per village**			**Number of households with small mammal captures per village**		
			
**Variable**	**n^a^**	**Median**	**Range**	**P value^b^**	**Median**	**Range**	**P value^b^**
**Number of households**				0.087			0.252
≤81	15	4	1–11		3	1–8	
>81	15	6	1–12		4	1–9	
**Drinking water**				0.181			0.226
Well	3	7	4–11		4	2–9	
Piped water	27	5	1–12		3	1–8	
**Houses raising chicken**				0.028			0.007
≤80%	6	6.5	5–11		5.5	4–9	
>80%	24	4	1–12		3	1–8	
**Rat control by chemical**				0.080			0.334
No	1	2	2		2	2	
Yes	29	5	1–12		4	1–9	
**Maize grown**				0.278			0.162
No	5	6	4–11		4	3–9	
Yes	25	5	1–12		3	1–8	
**Topography**				0.149			0.204
Mountain	11	4	1–12		2	1–6	
Basin among mountain	19	6	1–11		4	1–9	
**Communal latrine**				0.009			0.093
No	11	4	1–7		3	1–4	
Yes	19	6	1–12		4	1–9	

^**a **^Total number of villages is 30.

^**b **^P value from likelihood ratio test in univariate Poisson regression model.

Models for each of the 4 outcomes are shown in Table 4. The random effects component was not significant in the first three models and was therefore omitted, but was retained because of its statistical significance in the fourth model. The abundance ratios for number of small mammals captured and number of *R. flavipectus *are closely similar, consistent with the predominance of that species among captured animals. Greater numbers of small mammals and of *R. flavipectus *were captured in houses of the Dai ethnic group, where food was stored in metal drums rather than in sacks, wooden drums or other containers, where there was no outside toilet, where there were dogs but no cats, where there were adjacent houses and where there were no nearby rubbish dumps. At the village level, a greater number of household captures occurred in villages that had communal latrines and in those not growing maize.

**Table 4:** The adjusted abundance ratio (AR) or adjusted odds ratio (OR) for the four final models.

	**Total small mammals captures**		***R. flavipectus *captures**		***S. murinus *captures**		**Small mammal presence**	
				
**Variable**	**AR (95%CI)**	P value*	**AR (95%CI)**	P value*	**AR (95%CI)**	P value*	**OR (95%CI)**	P value*
**Ethnic group**		0.014		0.008				
Han and other	Ref **		Ref					
Dai	1.71 (1.10–2.66)		1.90 (1.15–3.14)					
**Type of food storage**		< 0.001		< 0.001		0.008		
Sack	Ref^ab^		Ref^ab^		Ref^a^			
Metal drum	2.31^c^ (1.40–3.80)		1.92^c^ (1.12–3.31)		8.46^b^ (1.71–41.89)			
Wood drum	1.39^b^ (0.90–2.15)		1.20^bc^ (0.76–1.91)		4.50^ab^ (0.96–21.21)			
Other	0.85^a^ (0.55–1.30)		0.58^a^ (0.36–0.94)		6.48^b^ (1.50–28.05)			
Keeping cat***		0.002		0.002				<0.001
No cat	Ref^a^		Ref^a^				Ref^a^	
Sleep in house	0.63^b^ (0.45–0.88)		0.62^b^ (0.43–0.90)				0.41^b^ (0.26–0.65)	
Not sleep in house	0.41^b^ (0.21–0.83)		0.31^b^ (0.13–0.72)				0.20^b ^(0.10–0.41)	
**Keeping dog**		0.029		0.002				
No	Ref		Ref					
Yes	1.42 (1.04–1.95)		1.76 (1.24–2.50)					
**Keeping duck**								0.037
No							Ref	
Yes							2.73 (1.01–7.37)	
**Surroundings – house**		0.002		0.010				0.011
No	Ref		Ref				Ref	
Yes	2.33 (1.29–4.23)		2.12 (1.13–3.96)				0.46 (0.25–0.85)	
**Vegetable grown near house**								
No					Ref			
Yes					0.27 (0.12–0.61)			
**Maize grown in village**		0.025		0.017				
No	Ref		Ref					
Yes	0.61 (0.40–0.93)		0.55 (0.34–0.88)					
**Rubbish dump near house**		0.006		0.011				
No	Ref		Ref					
Yes	0.64 (0.46–0.88)		0.63 (0.43–0.90)					
**Location of toilet**		0.016		0.041				
No toilet	Ref^a^		Ref^a^					
Inside house	1.17^a^ (0.67–2.05)		1.19^ab^ (0.63–2.22)					
Outside house	0.60^b^ (0.40–0.89)		0.61^b^ (0.39–0.94)					
**Communal latrine in village**		0.016		< 0.001				
No	Ref		Ref					
Yes	1.53 (1.07–2.18)		2.10 (1.37–3.23)					

* P value from likelihood ratio test in multilevel model.

** Reference category.

*** Adjusted for missing values.

^abc ^AR or OR not having a superscript in common within a variable and model are significantly different at p < 0.05 from Wald's test.

By contrast, greater numbers of *S. murinus *captures were associated with storing food in metal drums or other containers (mainly small covered baked-earth enclosures) compared with sacks, whereas fewer numbers were associated with growing vegetables adjacent to the house.

The presence of small mammals as evidenced by either the capture or reported sighting of small mammals in the last 2 weeks was more likely in households keeping ducks and less likely in household with surrounding houses. Keeping cats was associated with a lower probability of the presence of small mammals, similar to the findings of the models for small mammal capture.

## Discussion

Two species of mammal, *R. flavipectus *and *S. murinus*, were captured in the households of villages endemic for plague in Yunnan and the predictor profiles of each were somewhat different. Both species are known to be reservoirs for plague [14,15] and have been previously reported from southern China, including south-west Yunnan and the coastal areas of Zhejiang, Guangdong, Guangxi, Fujian, Hunan and Taiwan [16-18]. *R. flavipectus*, belonging to the order Rodentia, family Muridae, has been reported to be the main animal host and infectious source of plague in the commensal rodent plague foci in this region [19] and has been shown in surveillance data to be the dominant small mammal in Dehong prefecture, accounting for about 74% of small mammals captured [20], similar to the 80% in the current study.

*S. murinus*, belonging to the order Soricomorpha, family Soricidae, is reported to be an important reservoir of plague in Vietnam and Myanmar [18,21]. This species accounted for 19% of small mammals captured in earlier surveillance data, similar to the 20% in the current study.

Other species of small mammal have been occasionally trapped in surveillance operations, including *Mus caroli *and *Mus musculus *(order Rodentia, family Muridae), were not found in the current study, probably due to the small sample size.

Although previous studies have reported an inverse association between the abundances of *R. flavipectus *and *S. murinus *in Yunnan and Guangdong Province of China [17,22], no such association was evident in the current study. This may suggest somewhat different ecological niches in the current setting. This would be compatible with the somewhat differing predictor profiles of the two species. However, the small numbers of *S. murinus *captured resulted in low power to detect weak predictors of this species abundance, and this may account for some of the apparent difference in predictor profiles.

In any attempt to interpret the predictors of small mammals captured in traps it must be borne in mind that in order to capture an animal, it must first be present in, or pass by, the vicinity of the trap. It also must be susceptible to the lure of the bait, not afraid of the trap and not wise to the deception. In this study, traps and bait which have proved successful in routine capturing of small mammals in the same setting were used. However, it is likely that the effective lure that a bait presents depends not only on the nature of the bait itself but also on the state of hunger of the animal. Thus, predictors of mammal capture may relate to the presence of mammals in the house or to the likelihood of a mammal being attracted to the bait and succumbing to the deception. This may be the explanation of why more animals were caught in houses where food was stored in metal drums than in those where food was stored in sacks – the former presumably being considerably more small-mammal proof than the latter and therefore offering a less plentiful food supply, leading to a higher hunger level.

One of the most notable differences in the predictor profile between the two species is in the magnitude of the relationship between type of food storage and animal abundance. While storage of food in metal drums rather than in sacks appeared to favour the capture of both species, the effect was over four times greater in the case of *S. murinus*. Furthermore, other forms of storage – most commonly covered baked- earth enclosures – were associated with capturing fewer *R. flavipectus *but more *S. murinus*. This situation would be compatible with a situation in which the enclosures were penetrable by *R. flavipectus *but not by *S. murinus*. Given the differences in dentition of these two species – *R. flavipectus *adapted for gnawing and *S. murinus *for a mainly insectivorous diet [23-25] – this explanation has some biological plausibility.

The lower numbers of *S. murinus *caught in households around which vegetables were grown may also be an effect of the additional food supply for this species, which is reported to be an opportunistic feeder and whose diet includes plant material in addition to a wide variety of invertebrates and human food items [26]. Similarly, *R. flavipectus*, whose habitat is reported to include garbage dumps [27], may find a ready supply of food in garbage around the household as well as in maize plantations in the village and therefore have less interest in taking trap bait.

That these predictors of mammal capture act via the susceptibility to capture is also supported by the lack of discernable association with the sighting of small mammals in the household, a variable that is expected to be more closely representative of the presence of small mammals in the house.

Other aspects of the physical surroundings identified as being associated with higher numbers of *R. flavipectus *caught were location of the household adjacent to other houses and, at the village level, the presence of communal latrines. As *R. flavipectus *is a commensal rodent, an area of relatively dense housing might be expected to support a larger population of rats than an isolated house, and therefore the presence of adjacent housing is more likely to act via an influence on the numbers of rats rather than on susceptibility to capture. Relatively high levels of infestation have been reported in areas of high housing density with more than 500 dwellings in the immediate vicinity [28] and it was postulated that a nearby house might act as a source of rodent infestation, especially as the range of rodents may well encompass more than one house at a time and dispersal is more likely to be successful over a short distance. The home range and movements of small mammals in rural areas have been reported to be wider than in the urban habitat [29,30]. Our finding that location of the household close to other houses was associated with a lower probability of seeing small mammals, however, does not seem to be consistent with a higher level of infestation. Further investigation may be necessary to clarify this apparent anomaly.

The abundance of *R. flavipectus *was increased in villages with communal latrines. In general, latrines in rural areas are often semi-open and the sanitation is poor. This environment can offer shelter for rats and promote infestation. However, there seems to be some contradiction with the finding that households having an outside toilet had fewer rat captures. Further study of rat behaviour may be needed to better understand these relationships.

A cat in the house was associated with fewer rat captures and dogs in the house with greater numbers of rat captures. It is commonly understood that cats are important in the control of rats [28,31]. Sixty percent of households either kept cats or allowed cats from the neighbourhood to freely enter and sleep in the house. These households were also less likely to report seeing small mammals in the house, in accord with the finding of a previous study in southern Africa in which families that kept cats had a lower likelihood of seeing rodents than those without cats [32]. That study concluded that one cat could remove about 28 rats per year [31]. However, Childs (1986) has postulated that cats are ineffective in eradicating existing rat infestation because they kill mainly young rats [33]. Other studies have revealed that cats consume very few rats if food resources for the cats are plentiful [34]. Another study even found a positive relationship between the presence of cats and rats in urban areas, which was attributed to the common benefit – waste food [35]. There was no evidence that the presence of cats in the household affected the capture of *S. murinus*. It may be that the strong odour secreted by the well-developed scent gland of *S. murinus *deters potential predators [26].

Raising guard dogs is quite common in rural areas of Yunnan [4] and about one third of households raised at least one guard dog. Dogs were generally allowed to enter only the dining area and only in the daytime, in contrast to cats, that were allowed to roam freely throughout the house unchecked, and at any time. This difference may partially explain why the number of small mammals was not reduced by keeping a dog. In fact, the number of small mammals caught in households with a dog was increased. A similar finding was reported in urban areas of the USA where companion animals, especially dogs, were associated with the presence of rats because of the food and shelter provided by the pet owner [35].

Despite the identification of several predictors of *R. flavipectus *capture, other, as yet unidentified, lifestyle differences between the Dai and other, principally Han, ethnic groups, may also influence rat capture. Additionally, the reason for a higher probability of sighting of small mammals in households that keep ducks is not understood.

As discussed, numbers of animals trapped may reflect not only mammal population size but also susceptibility to capture, and trap setting for only two nights almost certainly underestimates the population size. The number of *S. murinus *captures was small and thus may not be powerful enough to detect some important predictors. Drawing conclusions regarding small mammal abundance from trapping results may need to be validated with other survey techniques. Furthermore, this cross-sectional study may be influenced by season and the results may change over the year.

## Conclusion

Small mammal captures were influenced by environmental conditions. Ready availability of food within and outside the houses was associated with fewer captures, especially of *S. murinus*, but appeared to have no effect on small mammal sightings. Presence of cats was related to reductions in both small mammal sightings and *R. flavipectus *captures. Other variables exhibited discrepancies between their associations with captures and those with sightings. These differences should be considered during the implementation and interpretation of small mammal surveys. Further international studies are needed to provide evidence of a cause-effect relationship between these predictors and the abundance of small mammals before the findings are applied directly to plague control measures.

## Methods

### Study design

A cross-sectional study was applied in this investigation. Field investigations were carried out in Lianghe county, Dehong prefecture, Yunnan Province, southwest China, between August and September 2007.

### Study setting

Lianghe county is one of 5 counties in Dehong prefecture bordering with Myanmar. In 2002, the total population was about 160,000 (89% of which were farmers). Ethnic groups include Han, Dai, A Chang, Jing Po, De Ang and others. Minority groups accounted for about 33% of the total population in this county. The average annual temperature is 18.3°C, average annual rainfall 1396.2 mm, and average annual sunshine 2385.5 hours. Economy mainly relies on agriculture. The average annual income per farmer in 2002 was 816 RMB (about US$100). Lianghe county was one of counties particularly supported by China central government because of poverty [36].

In 1990, a rat plague re-emerged in this county after a 33-year quiescent period. During the period of 1990 to 2006, among 381 villages of Lianghe county, 55 experienced at least one plague epidemic, 6 of them human and rodent plague and the others only rodent plague.

### Study villages and households sampling

Thirty-four villages were recorded as having had at least one rat plague epidemic in Lianghe county in the six years from January 2001 to December 2006. Four villages were not included because of difficult access. The remaining 30 villages were all farming communities, 11 located in mountainous areas and 19 in basin areas. The distance from each village to its closest neighbour in the sample varied from approximately 2 to 30 kilometres. Seventeen villages had experienced one epidemic in the six years, another 9 villages 2 epidemics, 2 villages 3 epidemics and 2 villages 4 epidemics.

A list of all households was obtained from the local village administration of each village. The median number of households in the villages was 81 (Range: 33 – 345). Eleven villages were unusually large and had subdivisions of village administration. In these villages, the subdivision with the highest number of households was selected to be the representative study unit. All households in each village were coded using a number starting from 1 to the total number of households. Twenty households per village were then randomly selected using a computer-generated random number algorithm.

### Survey for determinants of small mammal abundance

Household and village level data were collected using questionnaire and observation checklist at each level. At the village level, a face-to-face questionnaire-based interview was conducted with a purposively selected prominent and knowledgeable person in the village, such as the village doctor or head administrator. The interview covered the main source of economy, number of households and people, major ethnic group, drinking water source, presence of communal latrines, keeping of domestic animals, rat control measures and history of plague epidemic. The observation checklist included topography, major crops being grown, and presence and location of rubbish areas.

At the household level, the head of the household or the spouse, or both together, were interviewed face-to-face using a questionnaire covering details of main economic source, ethnic group, number of household members, highest education level among family members, food storage methods, waste disposal methods, place and type of toilet, keeping of domestic animals in the house, seeing "rats" in the house, having a rat problem and the practice of rat control. The observation checklist covered the type of house, surroundings of the house, crops grown adjacent to the house (within about 50 meters) and the presence of rat or other small mammal faeces.

These data were collected by the first author and other two trained interviewers from Yunnan Institute of Endemic Disease Control and Prevention (YIEDC). Each potential respondent was given a clear explanation of the research purpose and the respondent or other representative of the household asked to sign an informed consent form before interviewing and completing the checklist. Permission was obtained for placing traps to catch small mammals in the house.

### Small mammal trapping

Small mammal trappings in the houses were carried out by placing 5 live-traps (20 × 12 × 9 cm) along the walls or on rodent perceived runways in the bedroom, kitchen, main living room, utility room and store room for two consecutive nights. Three cages were baited with fried pork skin and two with fried wheat powder. Cages were set in the evening and checked in the morning of the following day. Location of trap and type of bait were the same on the 2 nights. Cages in which small mammals were trapped on the first night were replaced with new traps on the second night.

Small mammals captured were identified to species in the field according to their morphological features. Cages with captured small mammals were put into plastic bags and brought to the laboratory for collection of fleas. Results of the flea analysis are to be reported elsewhere.

### Statistical analysis

All data were coded and computerized using EpiData software [37] and analysed under R software [38]. Household- and village-level data were summarized using descriptive statistics.

The numbers of small mammals as well as the numbers of each species trapped per house were compared across categories of each household-level variable using tabulation and univariate random intercept Poisson models, in which village was considered to be a random effect variable. Numbers of households per village in which small mammals were trapped as well as the total numbers of small mammals trapped per village were compared across categories of village-level variables using both tabulation and univariate Poisson or linear regression models as appropriate.

Household- and village-level variables showing some evidence of a relationship (p < 0.2) with the magnitude of animals trapped and being plausibly potentially causal were selected for inclusion in initial multivariate random intercept multilevel Poisson models. Models were refined by a process of backward elimination of variables not contributing significantly to the fit using the change in log likelihood of successive models and p < 0.05 as the criterion for statistical significance. Finally the significance of the random component was evaluated. If not significant the model was re-developed omitting this random effects element. Coefficients of the variable in these models were exponentiated and defined as "abundance ratios".

As an alternative indicator of small mammal abundance in the study villages, the reported sighting of small mammals (commonly referred to locally as "rats") in the household within the previous 2 weeks, treated as a binary variable, was also analyzed. A new variable for small mammal presence was also created from the combination of small mammals seen or captured within the house. Tabulation, univariate random intercept logistic models, selection of candidate variables and development of a multivariate multilevel logistic model were performed using a strategy analogous to that described above for numbers of animals trapped. Coefficients from the model were exponentiated and defined as odds ratios.

In all models, missing values were accommodated by the method of covariate adjustment.

### Ethical approval

This research study was approved by YIEDC and the Ethics Committee of the Faculty of Medicine, Prince of Songkla University, before the research was carried out.

## Authors' contributions

JXY, AG, VC and XQD were responsible for conception and design of the study, JXY, XQD, CHD and YHZ supervised data collection, quality control of field work and data entry and checking. JXY, AG and EM analyzed and interpreted the data. JXY, AG and VC drafted and revised the paper. All authors read and approved the final manuscript.
